# Spiritual Needs Assessment in Post-Secular Contexts: An Integrative Review of Questionnaires

**DOI:** 10.3390/ijerph182412898

**Published:** 2021-12-07

**Authors:** Ricko D. Nissen, Erik Falkø, Tobias K. Stripp, Niels Christian Hvidt

**Affiliations:** 1 Research Unit of General Practice, University of Southern, 5000 Odense, Denmark; EFalkoe@health.sdu.dk (E.F.); tkstripp@health.sdu.dk (T.K.S.); nchvidt@health.sdu.dk (N.C.H.); 2 Academy of Geriatric Cancer Research, University of Southern, 5000 Odense, Denmark

**Keywords:** spiritual care, religion, spirituality, secular, post-secular, spiritual needs, assessment, questionnaire

## Abstract

Research across healthcare contexts has shown that, if provided appropriately, spiritual care can be of significant benefit to patients. It can be challenging, however, to incorporate spiritual care in daily practice, not least in post-secular, culturally entwined, and pluralist contexts. The aim of this integrative review was to locate, evaluate and discuss spiritual-needs questionnaires from the post-secular perspective in relation to their applicability in secular healthcare. Eleven questionnaires were evaluated and discussed with a focus on religious/spiritual (RS) wording, local culturally entwined and pluralist contexts, and on whether a consensual understanding between patient and healthcare professional could be expected through RS wording. By highlighting some factors involved in implementing a spiritual-needs questionnaire in diverse cultural and vernacular contexts, this article can assist by providing a general guideline. This article offers an approach to the international exchange and implementation of knowledge, experiences, and best practice in relation to the use of spiritual needs-assessment questionnaires in post-secular contexts.

## 1. Introduction

Research across healthcare contexts has shown that, if provided appropriately, spiritual care can be of significant benefit to patients, in terms of increased quality of life, better mental health, and lowered levels of anxiety and depression [1,2,3,4,5]. It can be challenging, however, to incorporate spiritual care in daily practice [5,6,7,8]. Reasons for this include lack of training or time, uncertainty regarding how to deliver spiritual care, confusion about the concepts of ‘spirituality’ and ‘religion’, and the often-difficult reflexivity of one’s own sense of spirituality, religiosity, or lack thereof [7,9,10,11]. Furthermore, the culturally entwined and pluralist context of the world [12] makes it essential to understand the existential/spiritual/religious grounding of the individual patient, and from there to develop the best possible approach to providing spiritual care [13].

The growing international focus on the positive potential of religion and spirituality in relation to health can be argued to be an expression of the post-secular, understood here as the realization of the continued presence of the religious and spiritual in the public sphere and the attempt to negotiate this presence in culturally entwined and pluralist contexts [14,15,16,17]. In healthcare, the post-secular can be seen in the now widespread consensus among policymakers, researchers, practitioners, and patients that integrating spiritual care at all levels of healthcare is both beneficial and recommendable [9,18,19]. The literature is teeming with research aimed at developing qualified approaches to providing spiritual care, such as tools for the assessment of spiritual needs, training in existential communication and spiritual care, frameworks for spiritual care, and international collaboration [20,21,22,23,24,25,26].

Spiritual-needs questionnaires arguably represent the most distributed intervention in relation to identifying spiritual needs [27]. However, the pluralist context makes it difficult to identify religious or spiritual needs through self-report questionnaires, because of the potential variance in spiritual and religious expression in any given context. Such variance can result in needs being reported predominantly on concepts relating to inner peace, generativity, and relatedness on personal levels, and to a lesser degree on religious and spiritual needs [28].

To address this situation, the aim of this integrative review was to identify spiritual needs questionnaires potentially applicable in a post-secular context and to evaluate and discuss them from the post-secular perspective in relation to their applicability in secular healthcare.

### Spiritual Care from a Post-Secular Perspective

We supplement the post-secular, as outlined above [14,15,16,29,30,31], with the understanding that post-secular contexts have been secularized at the macro level of societal discourses (such as healthcare) and are culturally entwined and pluralist at the level of the individual. By pluralist, and following Berger [12,17], we refer to both the co-existence of various religious, spiritual, and secularized existential orientations in society, and to how religious and spiritual individuals interact with secular societal discourses, such as economics, law, education, and, in this case, healthcare. Furthermore, we supplement this with the understanding that the process of secularizing societal discourses has, to various degrees, been accompanied by a privatization and individualization of existential thought—be it spiritual, religious, or secularized existential thinking [32,33]. This post-secular perspective allows for an understanding in which the presence of spiritual, religious, existential themes, thoughts, resources, and needs in the human being are recognized, appreciated, and brought into focus in the secular societal discourse of healthcare [34].

In our conceptualization of spiritual care, we focus on spiritual, religious, and secularized existential orientations, needs, and resources in connection with life-threatening illness and crisis [32,33,35,36,37]. Following this, ‘Spiritual Care’ is understood as an overarching concept through which existential, spiritual, and religious needs can be identified and addressed appropriately [13]. At the same time, we acknowledge that these concepts are constructions, and that individuals do not easily render themselves to such constructions [38]. Consequently, they may mean different things in different contexts and to different individuals. For these exact reasons, there is no international consensus on the definition of spirituality. In one context, a consensus on the definition of the concept may be reached quite easily, while in other contexts it may be an immature concept [39,40,41]. From the post-secular perspective, the vernacular used to identify spiritual and religious needs must be carefully considered and evaluated in relation to both local context and individual patient. How the constructs ‘the existential’, ‘the religious’, and ‘the spiritual’ are related to each other and how individuals understand themselves as spiritual, religious or neither spiritual nor religious, presents a very complex discussion. In the present study, we employ a simple understanding of religion as referring to institutionalized religion and related activities, and spirituality as a primarily subjective construct relating to ultimate questions and the meaning of life [32,42,43]. However, to address spiritual needs in post-secular contexts, which are culturally entwined and pluralist, we argue that these definitions are secondary to the patients point of view, which must be the primary focus when providing qualified and appropriate spiritual care. As expressed by Bash: “*spiritual experience is what each person says it is, and the task of nurses is to identify and respect that person’s expression of their spiritual experience and to offer them support.*” [44].

## 2. Method

This integrative review is based on the methodological approach suggested by Whittemore and Knafl [45]. The integrative review offers a stringent and transparent process of identifying, analyzing, and synthesizing results of studies on the same subject, while allowing for the inclusion of studies with various methodological approaches [46]. A comprehensive search of the literature was conducted with the initial aim of locating spiritual care instruments in general, in the databases PubMed, ATLA, and Scopus. The search string was defined for PubMed and refined to accommodate the specifications of the databases ATLA and Scopus. This search resulted in a scoping review, published as the Catalogue of Spiritual Care Instruments (CSCI) [27] (available online at https://faith-health.org/catalogue/, accessed on 15 October 2020). The CSCI includes 182 instruments, all applicable as interventions in the process of spiritual care [13]. The CSCI functions as the basis for literature for the present review.

**Figure 1:**
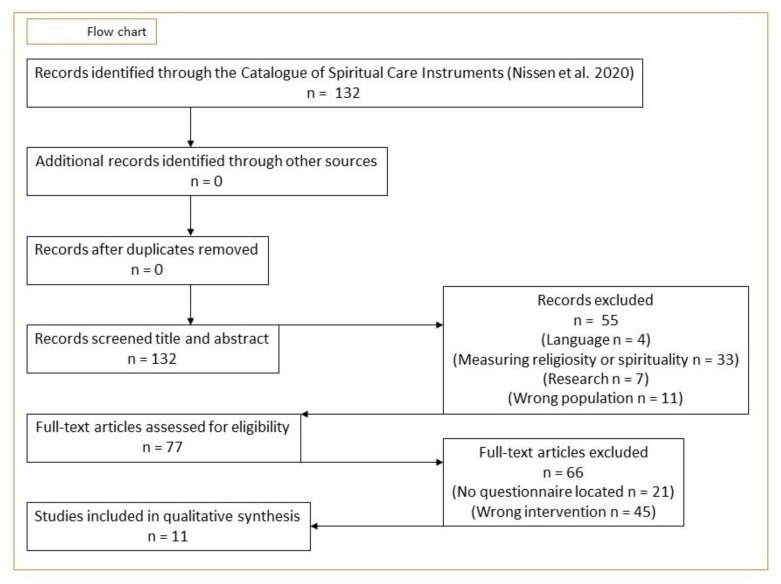
Flow chart.

In the present integrative review, an original research question and criterion for inclusion were developed, aimed at locating spiritual needs assessment questionnaires potentially applicable in culturally entwined and pluralist contexts/secular healthcare. The review was conducted in six steps. Step 1: Literature search and extraction of questionnaires from the CSCI (N = 132). Step 2: Evaluation of the questionnaires at title and abstract level, based on the criteria for inclusion (Table 1). This step was carried out independently by authors RDN and EF. Step 3: The remaining questionnaires (articles) (N = 77) were retrieved for full-text assessment, based on the inclusion criteria (Table 1). This step was carried out independently by authors RDN and EF. In case of conflict in the assessment for inclusion, the questionnaire was discussed in plenum with author NCH. Step 4: The remaining articles (N = 11) were discussed in plenum and their inclusion was decided upon based on consensus. Eleven questionnaires were included in the review. Step 5: clinimetric properties reported in the original validation of the 11 questionnaires were assessed. Step 6: Analysis, evaluation, and discussion of the included questionnaires. Figure 1 provides a flowchart of the described methodology.

**Figure 2:**
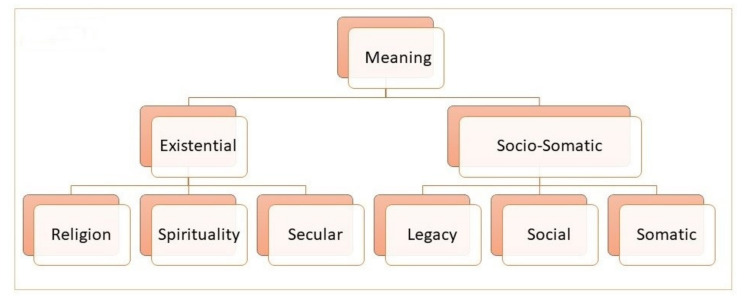
Inductive and taxonomic analyses.

The included questionnaires were coded and analyzed inductively [47,48,49]. Initially, the data was confronted with the question: What is this text about? From here, a coding frame was developed, and emerging categories were identified. The questionnaires were coded independently by authors RDN. and EF. The results were compared and discussed, and emerging categories, subdomains, and domains noted. The questionnaires were then re-coded on the basis of the consensus of established categories and to secure grounding in the data. A taxonomic analysis [49] established the final domain and subdomains, and these were discussed and fixed by consensus between the authors (Figure 2).

## 3. Results

Table 2 lists the 11 included questionnaires alphabetically and includes author, contextual origin, year of publication, primary health field, the Likert scale, and the number of questions included.

## 4. Inductive, Taxonomic Analysis, and Measurement Properties

Figure 2 shows the result of the inductive and taxonomic analyses, including six established categories, under two subdomains and under one domain.

The analyses established six categories. The categories ‘Religion’ and ‘Spirituality’ pertained to words that the author group agreed in plenum belonged to either a religious or a spiritual vernacular. These words are listed alphabetically in Table 3. We also provide the number of times the word appears across the questionnaires in Table 3.

The category ‘Secular’ (Figure 2) contains questions oriented towards existential topics and formulated in a secular vernacular. The ‘Legacy’ category contains questions oriented towards being remembered, having unfinished business, contribution to life, etc. The ‘Social’ category contains questions oriented towards social activities with family and friends. The ‘Somatic’ category contains questions oriented towards physical aspects in relation to illness, including questions directed to the healthcare professionals (HCP).

On the basis of these six categories, the taxonomic analysis established the sub-domains ‘Existential’ and ‘Socio-Somatic’ under the main domain ‘Meaning’, given that all the questions can be characterized as meaning-oriented, whether oriented towards making meaning in relation to religious, spiritual, existential, social, or somatic issues.

Table 4 shows the categories into which the questions in each questionnaire fall.

Table 5 provides an overview of the primary articles’ self-report on validation and clinimetric properties.

The instruments were evaluated based on the COSMIN Risk of Bias Checklist [61]. The checklist is not reported systematically here (Table 5), as we only report whether the authors of the questionnaires had succeeded in reporting the most common and appropriate clinimetric properties in their validation articles. The quality of the measures in themselves were not assessed. For the EOLSCQ, the authors failed to report how items were generated clearly and the original source for these could not be identified through online searches. An alpha of 0.98 was reported, indicating extremely high internal consistency, which demonstrates the risk of a high degree of redundancy between items. The reliability of FACIT-Sp-12 was reported by Brintz and colleagues [62], although this was not one of the papers identified through the review. The EDS’ clinimetric properties were reported after 20 participants had undergone qualitative interviews. The PSNAS had reported no formal validation methodologies, and the structure and items were generated based on theoretical assumptions.

Table 6 lists the religious/spiritual (RS) wording used in the respective questionnaires, the number of questions that contain RS wording and whether the RS wording used can be said to be neutral. Neutrality was evaluated from the question: Does the RS wording refer to a specific religious or spiritual orientation or not? The column ‘N of questions with RS wording’ does not necessarily match the ‘RS wording’ column, as one question may contain more than one RS wording, just as an RS wording may be used in more than one question in the same questionnaire.

## 5. Discussion: Evaluation of Applicability in a Post-Secular Context

It is imperative to note that the following evaluation is not to be considered as a final or conclusive evaluation as to whether any one of the questionnaires can be used in any given context. This is a general discussion that considers (some) of the topics that should be taken into account when implementing a spiritual-needs assessment questionnaire, viewed in relation to the post-secular perspective. It is for practitioners in local contexts to evaluate the extent to which a questionnaire, or part of a questionnaire, is applicable in the specific context and in relation to the particular patient in question [13].

It can be argued that all the included questionnaires represent examples of the post-secular in healthcare, as they assess patients’ spiritual, religious, and existential needs. Their use reflects an appreciation of the importance of addressing such needs in relation to patient health, while also, to varying degrees, contemplating the post-secular perspective of a culturally entwined and pluralist context. By further evaluating the individual questionnaires in relation to applicability in a post-secular context, we found that focusing on RS wording is the best method to approach such an evaluation (Table 6). We did not take into consideration how the authors defined the employed constructs and RS wording, nor the extent to which the various questionnaires employ subscales from other spiritual needs questionnaires. The focus here is on the post-secular perspective.

The number of questions employing RS wording in the included questionnaires ranges from 0 in the EDS to 10 in the QLQ-SWB-32 (Table 6). It would intuitively seem that the QLQ-SWB-32 would then be better at identifying spiritual needs than the EDS. However, this will depend on how the questions are formulated. Are they neutral in the RS wording, or do they refer to specific religious and spiritual orientations, thereby excluding others, and if so, why? The question (from PSNAS), “*At any time while you were in the hospital did you have a need: …To participate in religious or spiritual services?*” will identify a very specific need, namely the need to participate in religious/spiritual services, whereas a question (from FACIT-Sp-12) such as “*I find comfort in my faith or spiritual beliefs*” is more broadly formulated and identifies more generally whether there may be spiritual needs, which the HCP will then have to explore further. Both questions employ neutral RS wording, as they do not refer to any specific religious or spiritual orientation. Both originated in the USA (Table 2).

The contextual origin of a questionnaire will of course influence the way it is formulated and may explain why certain RS wording is used that refers to specific religious or spiritual orientations, while other religious or spiritual orientations are not referred to. The SNQPC, for instance, originated in Spain [59], where the predominant religious background is Catholicism. In 2017, 70.5% of the Spanish population considered themselves to be Catholic [63]. This is reflected in the SNQPC in the formulation of the RS wording, with questions such as “*Do you see your illness as a form of divine punishment or as a punishment for your life in general?*”, or “*Do you believe that God can intervene to cure a serious illness?*”. However, Spain is also a culturally entwined and pluralist context, with a secular state established in 1978 [63]. This raises questions about the dominant cultural background being based in a specific religious orientation. Where does this leave the pluralist condition, where the number of patients adhering to other religious, spiritual, or secularized existential orientations can be expected to increase? [12]. Furthermore, ethical questions need to be considered when using a question formulated as the above two examples, as to how the patient might be impacted by being asked such a question. A further reflection with respect to differences between the contextual origin of a spiritual needs questionnaire is whether the formulation of the questions reflects constitutional differences regarding the pluralist condition. The pluralist condition, as formulated in the First Amendment of the US Constitution, where equality between religious denominations is ensured by preventing the state from creating or favoring a religion [64], differs significantly from the pluralist condition as formulated in many European contexts, where a national church often holds a favored position in the constitution but also in relation to identification and tradition in the general population [13]. The Constitutional Act of Denmark, for instance, states that the established church of Denmark is the Evangelical Lutheran Church and shall as such be supported by the state (§4) [8].

It is not feasible to refer to and include all religious and spiritual orientations; however, from the post-secular perspective, this raises the following question: when all religious or spiritual orientations cannot be mentioned, why mention even one? In culturally entwined and pluralist contexts, spiritual needs questionnaires should consider including, instead of excluding, religious, spiritual, and secular existential orientations. Especially so if the questionnaire is formulated as, or applicable as, a self-report questionnaire. These are complex considerations that highlight the importance of the local context to explicitly contemplate the cultural and pluralist complexity of the context and of the (patient) population. The six questionnaires that we evaluated as using exclusively neutral RS wording (Table 6) originate from Canada (EDS, PDI), Portugal (EOLSCQ), and the USA (FACIT-Sp-12, MiLS, PSNAS). The two remaining questionnaires originating in European contexts, SNQPC and SpNQ, both reflect the dominant Christian contexts in Spain (SNQPC) and Germany (SpNQ). The QLQ-SWB-32 was developed by the European Organisation for Research and Treatment of Cancer (EORTC) Quality of Life Group and is an attempt at developing a spiritual well-being tool, applicable in multiple cultural and linguistic contexts [58]. The workgroup also included collaborators from contexts outside the EU (China, Chile, Singapore, Japan, Mexico, and Iran). The majority of the questions in the QLQ-SWB-32 (N = 22) are formulated within the categories ‘Secular’, ‘Legacy’, and ‘Social’ (Figure 2 and Table 4). The question “*I believe in life after death*”, is evaluated as not being neutral, as it refers to religious or spiritual orientations that include the concept of life after death. The concept of God, also evaluated as non-neutral, is used three times without reference to any specific God. However, when formulated in the singular, the monotheistic God of Judaism, Islam, and Christianity does come to mind, and not the plurality of gods from, e.g., Hinduism, or non-theistic orientations such as Buddhism. The HHSQ, which is from Hong Kong, is the only questionnaire that refers to religious traditions from both monotheistic religions and the religious orientations employing the concept of Karma. This may reflect an approach to the pluralist context in Hong Kong. The QEHS (New Zealand) was developed as a holistic measure whereby the existential, spiritual, and somatic aspects are seen as integrated, as illustrated in the question, “*In the past week, how frequently did you …* *Look after your spiritual, emotional, and mental self, and find your physical condition was also better?*”. This holistic integration of the spiritual and somatic brings attention to the limitations of the constructs we employ and how human beings do not necessarily comply with these constructs [38,65]. From the perspective of New Zealand and other post-colonial contexts, a further nuance is the relation to indigenous cultures and debates about culturally appropriate healthcare [66], which could provide a perspective on QEHS for incorporating the holistic approach.

In considering whether a spiritual-needs questionnaire with minimal RS wording suffices in identifying spiritual needs, the question arises as to whether HCPs can successfully identify religious or spiritual needs on the basis of the questionnaire. Using questionnaires with a primarily neutral RS wording can reach a wider patient population. However, this leaves the responsibility of identifying religious or spiritual needs to the HCP. Whether the involved HCPs are adequately trained in evaluating and identifying spiritual needs should be considered, as well as what the appropriate interventions might be, and how to proceed in developing a spiritual care treatment plan that appropriately and ethically addresses the specific needs [13]. The importance of training and educational programs in spiritual care is stressed as important in the literature, and many contexts have now implemented spiritual care in the curriculum and training has been developed for providing spiritual care [67,68,69,70]. However, research also stresses that spiritual care is often left to ad hoc or arbitrary solutions and linked to personal values [71,72].

In examining questionnaires that include RS wording that is not neutral, we argue that a consensual understanding of RS wording needs to be established between patient and HCP. The RS wording ‘spirituality’ may serve as an example. We classify spirituality as neutral RS wording because it does not refer to a specific spiritual orientation. However, and as mentioned earlier, spirituality (and through that spiritual care) is an ambiguous term because it is a construct that lacks consensus with regard to its definition, in both local and international contexts [36,41,73]. In one context, a common consensus on what the word entails may have been developed, while in other contexts it may be an immature concept [39,40]. From the post-secular perspective, even an apparently neutral concept such as ‘spirituality’ may be difficult to assess, and spiritual care needs to be addressed and considered in spiritual-needs questionnaires. Whether the patient and HCP can be expected to have a shared understanding on the meaning of RS wording needs to be evaluated. In pluralist settings, it might be challenging to reach a consensus on wording. The use of non-neutral RS wording may assist in developing a dialogue between patient and HCP in the attempt to reach a consensual understanding and thereby identify needs. These factors should also be taken into account in the translation of questionnaires [74]. The consideration of the culturally entwined context has been identified as central in research [75,76]. However, the process of translation does not necessarily account for the importance of a consensual understanding of RS wording. We argue this to be imperative, if spiritual care questionnaires are to be employed in post-secular contexts that have different local understandings of RS wording.

Finally, a brief comment on the category of secular questions, the inclusion of somatic questions, and healthcare field (Figure 2 and Table 4) is provided. Secular-formulated questions can identify existential needs. However, whether they are able to identify religious or spiritual needs will depend on whether the HCP identifies that religious or spiritual needs may be at the root of the answers given by a patient, and therefore requires further clarification, as argued above. In relation to including somatic questions with existential, religious, or spiritual questions, this should be considered from an ethical perspective, as to whether it is appropriate to have questions pertaining to, for instance, personal hygiene, next to existential, religious, and spiritual questions. The HHSQ provides 45 questions with 21 in the socio-somatic domain and 24 in the existential domain. This reflects the fact that the HHSQ is a holistic health assessment questionnaire. This leads to considerations of how many questions are needed and how many questions the patient is capable of answering. This point has to be seen in relation to the diagnosis and health situation of the patient, which also includes considerations about the healthcare field in which the questionnaire has been developed. The questionnaires included in this review are focused on end-of-life, palliative care, cancer, and chronic illness (Table 2). Only the local context can evaluate these considerations, bearing in mind that it is the well-being of the patient, both physical and existential, that lies at the heart of healthcare.

## 6. Conclusions

In conclusion, this integrative review has focused on identifying spiritual-needs questionnaires that could be applicable in secular healthcare in post-secular contexts. Eleven questionnaires were included for evaluation, which was conducted on the basis of inductive and taxonomic analyses. The evaluation and discussion focused on the religious and spiritual words used in the questionnaires, highlighting various topics that need to be addressed when evaluating the applicability of a spiritual-needs questionnaire in a post-secular context.

The evaluation focused on factors to be considered when implementing a spiritual-needs questionnaire in a post-secular context, mainly the pluralist condition, the use of RS wording, and a reflection on the originating context.

The findings provide a perspective for healthcare fields in which spiritual needs questionnaires are to be translated from a different cultural context. By highlighting some of the factors that should be taken into consideration, this article can act as a general guideline for local contexts when developing approaches to identifying spiritual needs and providing spiritual care that is applicable in daily practice.

From these perspectives, this article offers an approach to the international exchange and implementation of knowledge, experiences, and best practice in relation to the use of spiritual needs assessment questionnaires in post-secular contexts.

## Figures and Tables

**Table 1:** Criteria for inclusion.

Inclusion: The Instrument Had to Be (Listed Alphabetically)
A spiritual needs assessment questionnaire or applicable as such
Applicable as a self-report questionnaire
Any of the following target groups: chronic disease patients, life-threatening illness, or terminal patients (end-of-life)
Deemed applicable in a post-secular context, by initial face validation
Published in a peer-review journal
Written in English

**Table 2:** Questionnaires.

Instrument Name Abbreviation	Author, Origin Year	Health Field	Likert Scale	Number of Questions
Existential Distress Scale EDS	Lo et al. Canada 2017 [50]	Cancer	5	10
(Portuguese) End of Life Spiritual Comfort Questionnaire EOLSCQ	Pinto et al. Portugal 2016 [51]	EOL/PC	6	28
Functional Assessment of Chronic Illness Therapy —Spiritual Well-Being Scale FACIT-Sp-12	Peterman et al. USA 2002 [52]	Cancer—Chronic illness	5	12
Holistic Health Status Questionnaire HHSQ	Chan et al. Hong Kong 2016 [53]	Chronic illness	4	45
Meaning in Life Scale MiLS	Jim et al. USA 2006 [54]	Cancer	6/4	21
Patient Dignity Inventory PDI	Chochinov et al. Canada 2008 [55]	EOL/PC	5	25
Patient Spiritual Needs Assessment Scale PSNAS	Galek et al. USA 2005 [56]	EOL/PC	6	29
QE Health Scale QEHS	Faull & Hill, New Zealand 2007 [57]	Chronic physical disabilities	5	28
Quality of Life Questionnaire—Spiritual Wellbeing—32 QLQ-SWB–32	Vivat et al. EU 2017 [58]	EOL/PC	4/8	32
Spiritual Needs Questionnaire for Palliative Care SNQPC	Vilalta et al. Spain 2014 [59]	Cancer/PC	5	28
Spiritual Needs Questionnaire SpNQ	Büssing et al. Germany 2010 [60]	Cancer—Chronic illness	Y/N/3	27

**Table 3:** List of religious or spiritual words used in the included questionnaires.

RS Word	N	RS Word	N	RS Word	N	RS Word	N
Angel	1	Fate	1	Pastor	1	Religion	11
Allah	1	God	8	Pilgrimage	1	Ritual	1
Christian resurrection	1	Heaven	1	Power outside yourself	1	Sacrament	1
Divine intervention	1	Higher presence	1	Pray	7	Saint	1
Divine punishment	1	Karma	1	Predestined	1	Spirituality	24
Faith	9	Life after death	1	Reincarnation	1		

**Table 4:** Existential and socio-somatic sub-domains.

	Existential				Socio-Somatic			Total
Instrument	R	S	RS	SEC	L	SOC	SOM	
**EDS**	0	0	0	6	1	3	0	10
**EOLSCQ**	1	0	0	13	0	4	10	28
**FACIT-Sp-12**	0	0	3	8	1	0	0	12
**HHSQ**	5	1	0	18	1	7	13	45
**MiLS**	0	0	3	18	0	0	0	21
**PDI**	0	1	0	13	2	4	5	25
**PSNAS**	1	1	3	15	2	4	3	29
**QEHS**	0	2	1	19	0	4	2	28
**QLQ-SWB-32**	2	1	7	16	1	5	0	32
**SNQPC**	6	1	0	8	5	4	4	28
**SpNQ**	6	0	1	8	3	9	0	27

R = religious, S = spiritual, RS = religious/spiritual, SEC = secular, L = legacy, SOC = social, SOM = somatic.

**Table 5:** Clinimetric properties self-reported in primary articles.

Instrument	Items Selected Based on Interviews	Content Validity	Construct Validity	Reliability	Internal Consistency	Measurement Error	Responsiveness
**EDS**	+	+	+	-	+	-	-
**EOLSCQ**	-	+	+	-	+	-	-
**FACIT-Sp-12**	+	+	+	+	+	-	-
**HHSQ**	+	+	+	+	+	-	-
**MiLS**	-	+	+	+	+	-	-
**PDI**	+	+	+	+	+	-	-
**PSNAS**	-	-	-	-	-	-	-
**QEHS**	+	+	+	+	+	-	-
**QLQ-SWB-32**	+	+	+	+	+	-	-
**SNQPC**	+	+	-	-	-	-	-
**SpNQ**	+	+	+	-	+	-	-

**Table 6:** Secular applicability.

Instrument	RS Wording	N of Questions with RS Wording	Neutral
**EDS**	0	0	Yes
**EOLSCQ**	Faith	1	Yes
**FACIT-Sp-12**	Faith, Spiritual	2	Yes
**HHSQ**	Divine intervention, Fate, God, Heavens, Karma, Pray, Predestined, Religion	6	No
**MiLS**	Faith, Spiritual	2	Yes
**PDI**	Spiritual	1	Yes
**PSNAS**	Power outside yourself, Pray, Ritual, Religious, Spiritual	5	Yes
**QEHS**	Faith, God, Spiritual	3	No
**QLQ-SWB-32**	God, Life after death, Meditation, Pray, Spiritual	10	No
**SNQPC**	Christian resurrection, Divine punishment, Faith, God, Pilgrimage, Reincarnation, Religious, Sacrament	7	No
**SpNQ**	Allah, Angels, God, Higher presence, Life after death, Pastor, Pray, Religious, Saints, Spiritual	7	No
